# Blocking transmission of vector-borne diseases

**DOI:** 10.1016/j.ijpddr.2017.01.004

**Published:** 2017-01-30

**Authors:** Sandra Schorderet-Weber, Sandra Noack, Paul M. Selzer, Ronald Kaminsky

**Affiliations:** a Sablons 30, 2000 Neuchâtel, Switzerland; b Boehringer Ingelheim Animal Health GmbH, Binger Str. 173, 55216 Ingelheim, Germany; c ParaC Consulting for Parasitology and Drug Discovery, Altenstein 13, 79685 Haeg-Ehrsberg, Germany

**Keywords:** Vector-borne diseases, Transmission blocking, Drug discovery, Speed of kill

## Abstract

Vector-borne diseases are responsible for significant health problems in humans, as well as in companion and farm animals. Killing the vectors with ectoparasitic drugs before they have the opportunity to pass on their pathogens could be the ideal way to prevent vector borne diseases. Blocking of transmission might work when transmission is delayed during blood meal, as often happens in ticks. The recently described systemic isoxazolines have been shown to successfully prevent disease transmission under conditions of delayed pathogen transfer. However, if the pathogen is transmitted immediately at bite as it is the case with most insects, blocking transmission becomes only possible if ectoparasiticides prevent the vector from landing on or, at least, from biting the host. Chemical entities exhibiting repellent activity in addition to fast killing, like pyrethroids, could prevent pathogen transmission even in cases of immediate transfer. Successful blocking depends on effective action in the context of the extremely diverse life-cycles of vectors and vector-borne pathogens of medical and veterinary importance which are summarized in this review. This complexity leads to important parameters to consider for ectoparasiticide research and when considering the ideal drug profile for preventing disease transmission.

## Highlights

• Novel drugs against ectoparasites should prohibit vectors to transmit pathogens.
• Timing events of most human and animal vector-borne diseases are reviewed.
• Most ticks and insects show substantial differences in transmission time.
• Drug profile requirements differ between ticks and insects.
• Ideal new ectoparasiticides combine fast speed of kill and repellency.

## Graphical abstract

**Figure:**
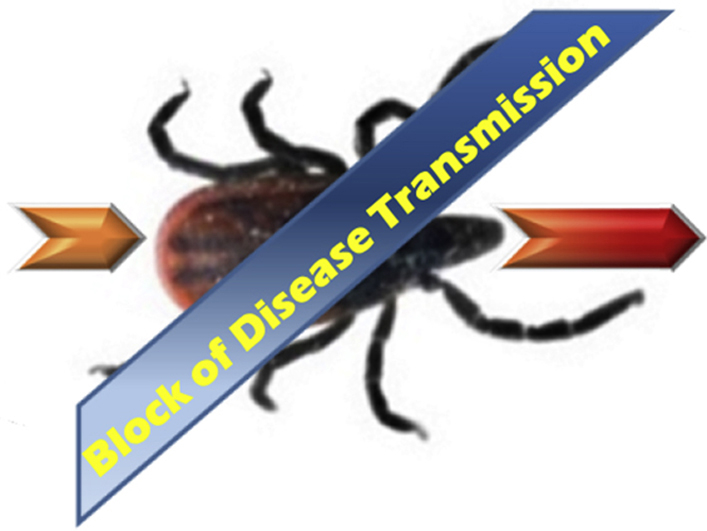

## 1 Introduction

**Fig. 1:**
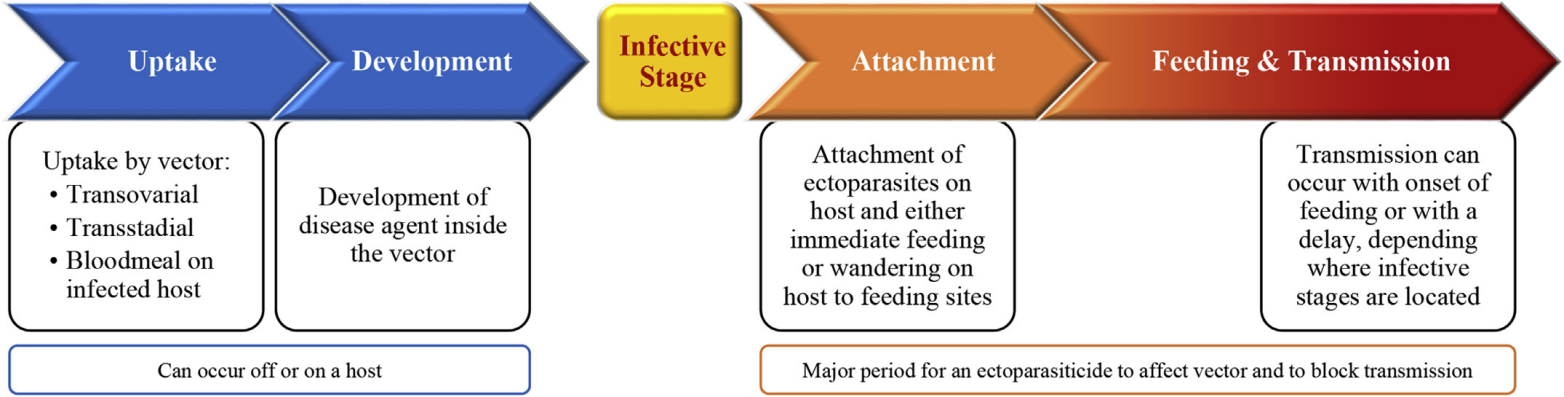
Generic sketch for transmission of diseases by ectoparasites (vectors). Blocking of transmission can in principle occur at every stage, but most drugs aim to interfere during “Attachment” phase and/or “Feeding & Transmission” phase.

Blood-feeding ectoparasites are responsible for severe aggravation through their constant attempts to get blood from their hosts. Besides causing discomfort, allergic reactions, skin damage and pain, many ectoparasites are also vectors of life-threatening or debilitating diseases caused by the transmission of a wide variety of pathogens, i.e. viruses, bacteria, protozoans, and worms, adding to their economic and emotional impact on human and animal health (Mehlhorn, 2008). Therefore, requirements for new ectoparasitic drugs should include not only the control of ectoparasites for a certain period of time, but also address their ability to block the transmission of the various vector-borne pathogens by a rapid onset of action. In this scope, “speed of kill” has become an important commercial differentiator for recent marketed products (Halos et al., 2014, Wengenmayer et al., 2014, Beugnet et al., 2016, Blair et al., 2016, Six et al., 2016) and many studies have been designed for testing the ability of those products to block transmission of some important pathogens of cats like *Bartonella henselae* (Bradbury and Lappin, 2010), and of dogs like *Dipilidium caninum* (Fourie et al., 2013a), *Leishmania infantum* (Brianti et al., 2014), *Ehrlichia canis* (Jongejan et al., 2015), *Borrelia burgdorferi*, *Anaplasma phagocytophilum* (Honsberger et al., 2016), and *Babesia canis* (Beugnet et al., 2014, Taenzler et al., 2016). These studies all report a complete prevention of pathogen transmission by fast elimination of the vector. These promising results confirm that a rapid onset of action should be an essential component of a novel drug profile. However, due to the diversity and specificity of vector parasite interactions, the blocking characteristics of those ectoparasiticides may not be sufficient to control other major pathogen transmitted by vectors to human, companion and farm animals. The arthropod can be either a mechanical vector, that is a simple carrier for dispersion, or a biological vector, within which the pathogen undergoes asexual and/or sexual multiplication before being transferred to a mammalian host. In the latter situation, pathogens need time to undergo development inside the vector and reach their infective stage. This depends to a major part on environmental conditions like temperature and humidity, and on the ability of the vector to survive long enough to harbor the matured infectious stage to be transmitted at next bite. Blocking pathogen transmission during that period has been tried with success as seen with *Ixodes scapularis* and *Borrelia burgdorferi* (Eisen and Dolan, 2016). Treating only the mammalian host with an efficient drug is a simpler option, but ensuring that a new drug is able to reliably block pathogen transfer remains very challenging. Nevertheless, there is a window of time for an ectoparasitic drug to prevent disease transmission (Fig. 1). This time period differs in length for each pathogen and vector, and can last from mere seconds to weeks.

Here we catalog a fair number of ectoparasite vectors and the respective transmitted pathogens of medical and veterinary importance. In addition, we complement that list with published information on the pathogen transmission time. Based on these results we propose several characteristics for an effective ectoparasitic drug profile.

## 2 Vector-borne transmitted pathogens

Major pathogens of medical and veterinary importance are listed in Table 1, Table 2, Table 3. A short description of their development in the vector and timing of transmission is given when available. For each category of mammalian hosts, vectors are listed according to their importance in disease transmission. Many pathogens are zoonotic, with companion or farm animals becoming reservoirs in close contact with human populations, thus highlighting the practicality of employing common strategies for both human and animals to control pathogen transmission. The tables demonstrate the diversity of the vector – pathogen interactions. In most cases, the pathogen will undergo a multiplication, a change in morphology, and a maturation in the vector. Very often, the infectious pathogen is waiting in the vector's salivary glands and will be passed on to the host together with the saliva immediately at bite. On the other hand, there are a few organisms, like *Rickettsia* sp (Hayes and Burgdorfer, 1982), *Anaplasma* sp (Hodzic et al., 1998, Katavolos et al., 1998, des Vignes et al., 2001), *Borrelia* sp (Kahl et al., 1998, des Vignes et al., 2001), or *Babesia* sp. (Homer et al., 2000, Zintl et al., 2003), that need an activation step for migration into the salivary glands, multiplication within the salivary glands, or a maturation phase all triggered by the onset of the blood meal. Interestingly, these pathogens all mature in ticks, which are slow blood-feeding arthropods and typically need days of host attachment to fully engorge.

## 3 Transmission time is considerably different between insects and ticks

When considering ectoparasites in relation to the pathogens transmitted and the time needed to transfer the pathogens after biting the host, a clear difference between insects and ticks is noticeable (Table 1, Table 2, Table 3). Many, if not all holometabolic insects like mosquitoes (Vlachou et al., 2006), tsetse flies (Van Den Abbeele et al., 1999), fleas (Gage and Kosoy, 2005), or sand flies (Bates, 2007), which undergo complete metamorphosis, almost always transfer the respective pathogens immediately at bite. By contrast, some ticks can require host attachment time periods of several hours, extending up to days in some instances before transmission of pathogens occurs. As hard ticks (*Ixodidae*), sometimes also referred to as hardbacked ticks, feed only once before molting to the next stage, ingested pathogens will have to survive the molting process and be transferred transstadially (i.e. *Babesia* sp, Homer et al., 2000; *Ehrlichia* sp, Paddock and Childs, 2003, Stich et al., 2008). It may be difficult for the pathogen to develop, migrate, or mature while the physical and metabolic changes take place during the vector's molting process. The pathogen will also have to survive for an extended period in the tick vector that might not find the next host immediately and could stay unfed for weeks or months. Those micro-organisms may then need a reactivation from some kind of dormant condition to resume their development. Temperature change due to the tick attachment to a warm-blooded animal (Hayes and Burgdorfer, 1982), or fresh blood entering the tick may be the signal for the pathogen to multiply (Hodzic et al., 1998), migrate to salivary glands (Kahl et al., 1998), or finish its maturation and be ready for transmission (Homer et al., 2000). This last step might take hours or days, and gives opportunities to block the transfer. In this context, fast-acting ectoparasiticides could be effective at preventing disease transmission in ticks (Reichard et al., 2013, Fourie et al., 2013a, Brianti et al., 2014, Jongejan et al., 2015, Beugnet et al., 2014, Honsberger et al., 2016, Taenzler et al., 2016).

In soft ticks (*Argasidae*), also referred to as softbacked ticks, pathogens face similar conditions as in hard ticks (i.e. survival through molting, long periods of fasting, transstadial transmission) but also have to adapt to additional constraints. Soft ticks like *Ornithodoros* are fast blood-feeders that need only minutes to fully engorge. Adults feed many times, and females lay eggs in small batches after each blood meal. They develop through more than one nymphal stage, increasing the number of opportunities for transmitting pathogens during their life-span (Schwan and Piesman, 2002). Fast-feeding implies that pathogens cannot go through an activation step during the blood meal like that previously discussed for hard ticks, but rather have to be ready in the salivary glands to be transferred as soon as feeding starts. As an example, *Borrelia duttoni* infecting soft ticks is transmitted from within 30 s to a few minutes after feeding starts (Dworkin et al., 2008), whereas *B. burgdorferi* is only transmitted by hard ticks after 24 h–48 h on average (des Vignes et al., 2001, Schwan and Piesman, 2002). Thus a drug with an onset of action within a few hours might be sufficient for blocking transmission by hard ticks, but not for preventing transmission by soft ticks. In the latter case, preventing the vector from accessing the host with a repellent could be a more effective solution.

Some major pathogens of hemimetabolic insects like true bugs (*Reduviidae*) or lice (*Phthiraptera*) can develop in either immature stages or adults. *Trypanosoma cruzi* (Krinsky, 2008), *Rickettsia prowazekii* (Houhamdi et al., 2002), or *Bartonella quitana* (Byam and Lloyd, 1920) are transmitted to their host via infected feces rubbed on wounded skin. Killing the vector before it gets time to produce infected feces could be possible using a drug with very fast onset of action. In the case of lice, such a drug could also have a massive impact on lice populations that do not move easily from one host to another, and therefore reduce the inflammation and scratching that are the real cause of infection. In *Reduviidae*, blocking transmission via killing the insect before releasing infected feces may also work. However, as *Reduviidae* are fast feeders, release of the feces could occur within the first minutes of a blood meal. It remains to be demonstrated if preventing access to a host and subsequent biting with a repellent drug can effectively block *T. cruzi* transmission.

Pathogens of most holometabolic insects develop and multiply in an adult individual that has a life expectancy on the order of days or weeks. Their development can start immediately after ingestion and needs to reach the infective stage within the life-span of the insect vector. In these cases the pathogen strategy appears to be different and infectious stages are transmitted often within seconds to the mammalian host. Blocking transmission is therefore more challenging, and avoiding insect bite via a repellent drug could be the best option.

## 4 Drug profile for blocking pathogen transmission

The principal feature of an ectoparasitic drug aiming to block transmission should certainly be a very fast onset of action. This requirement is generally understood by the animal health industry, and most products marketed recently have been tested and compared for the speed of their onset of action (Halos et al., 2014, Wengenmayer et al., 2014, Beugnet et al., 2016, Blair et al., 2016, Six et al., 2016). Recent compounds deriving from the fairly new chemical class of isoxazolines (Weber and Selzer, 2016) exhibit their ectoparasitic action against both, insects and acari of veterinary importance, within hours, and certainly reduce the risk of disease transmission of hard tick pathogens that are not immediately passed on to the host such as *Babesia* sp (Beugnet et al., 2014, Taenzler et al., 2016) or *Borrelia* sp. (Honsberger et al., 2016). Such a beneficial effect was shown especially for canine borreliosis (Honsberger et al., 2016, Weber and Selzer, 2016). Based on those results, one could hypothesize that isoxazolines may also be able to prevent human borreliosis (Lyme disease). However, to date many unknowns remain, including the pharmacokinetic behavior and safety of the drug in humans. Although effective at eliminating some tick infestations and consequently blocking pathogen transmission, systemic ectoparasiticides may be more limited in controlling those pathogens that are transmitted within a few hours or immediately after the vector's bite. For example, in a comparative study on the ability to block *Ehrlichia canis* transmission from *Rhipicephalus sanguineus* ticks by orally administered isoxazolines compared against topically applied products containing synthetic pyrethroids, the tested systemic isoxazoline ectoparasiticides gave insufficient protection of dogs from pathogen transfer (Jongejan et al., 2016). Despite being considered “old drugs”, synthetic pyrethroids (i.e. permethrin, deltamethrin, flumethrin) exhibit features that would in principle be close to an ideal drug profile. In addition to having a fast onset of action on many insects and tick species, some pyrethroids are also irritant or repulsive for a variety of ectoparasites (Mencke, 2006). It appears that a combination of repellency and parasiticidal activity could be the best way to prevent pathogen transmission, independently from the transfer time at bite. Synthetic pyrethroids have been shown to efficiently block transmission of *Leishmania* sp. in dogs by repelling and killing sandflies (Ferroglio et al., 2008, Brianti et al., 2014). They are also widely used for impregnating bed nets and clothing to prevent insect bites and disease transmission to humans (Curtis et al., 2003, Banks et al., 2014). They have been added to some recently marketed products for companion animals, to act as repellents and/or speed up the onset of action (Beugnet et al., 2016, Blair et al., 2016). However, wide-spread resistance in many vectors (including mosquitoes, lice, true bugs, and ticks) and safety issues (Anadón et al., 2009, Peterson et al., 2011) disqualify them for longer-term use and motivate the search for novel drugs displaying an equivalent profile with improved safety. Designing and developing new and safe ectoparasiticide drugs able to effectively block fast transmitted vector-borne pathogens is still on a wish list and remains extremely challenging. In our opinion, such novel ectoparasitic drug for animal health, should combine features of fast killing, long persistency and repellency to both acari and insects. Additional constraints may be encountered if any new ectoparasiticide should be considered for human use. Beyond identifying a relevant application, it is not clear if humans would accept a persistent drug exposure to achieve a long lasting protection period. In principle, repellency combined with long-term persistence is very difficult to achieve in a single compound, constituting a challenge as big as achieving very rapid onset of action. In addition, a drug with only repellent activity would have the disadvantage of having no impact on vector populations. The fast killing and long lasting persistence already achieved with the isoxazolines would allow prevention of important tick-borne diseases. Additional repellency or deterrent activity would be efficacious at preventing insect-borne pathogens that are transmitted rapidly upon biting. Combining all of these activities would be the ideal profile for an ectoparasiticide. Achieving that goal might not be possible with a single chemical entity but may be possible with a combination of molecules, bearing in mind the challenges of maintaining a good safety profile for the host and for the environment. Hurdles remain extremely high however, and other complementary measures targeting the pathogen itself via specific drugs or vaccines should definitely be investigated in parallel.

## 5 Conclusion

Meeting the requirements for new ectoparasiticides, including prevention of transmission of pathogens, is challenging if possible at all. Transmission in companion animals of some major tick-borne pathogens can be now controlled with compounds of the isoxazoline class because of their fast onset of action. Extending the use of this class of molecules to humans and farm animals may help to control some tick-borne zoonotic diseases. For other pathogens, mainly those transferred to the host by insects immediately at bite and by soft ticks, the speed of kill by isoxazolines is insufficient to effectively prevent pathogen transmission. Most insect vectors have little time for feeding before being chased away or being killed by the host, and therefore, in most cases, blood feeding and associated pathogen transmission begins immediately upon landing. In this situation, drugs having repellent or deterrent activity that hinders the vector from biting or landing on the host would be more successful at preventing disease transmission. Solutions could, therefore, be different depending on the vector, the associated pathogens and the speed of transmission. In an ideal situation, a drug or a combination of chemical entities should prevent the vector from access, or at least from biting the host. If the vector eventually succeeds in reaching the host, killing by the drug should happen very rapidly. Repellent efficacy combined with parasiticidal activity seems to be the ideal drug profile for successfully preventing vector-borne diseases in humans, pets and livestock. This easy statement unfortunately hides major difficulties especially if the repellent effect has to be long-lasting for weeks or months. Due to those substantial difficulties, the search for new vaccines or drugs targeting the pathogen should not be left aside. Novel alternative approaches, for example ones based on regulators of the immune system like the Toll pathway of the vector (Garver et al., 2009) should also continue to be explored.

## Figures and Tables

**Table 1:** Human vector-transmitted pathogens (*zoonotic diseases).

Vector	Pathogen class		Disease	Pathogen development phases and timing in vector	Pathogen- to- host transfer timing at vector bite	References
Mosquitoes (Culicidae) *Aedes* sp *Culex* sp *Anopheles* sp *Ochlerotatus* sp	Arboviruses	Togaviridae	Chikungunya, Ross River	Replication occurs first in midgut cells, followed by dissemination to other organs including salivary glands with additional multiplication cycles. Development success in vector is dependent on temperature, vector competence, and viral dose at infection. An EIP (extrinsic incubation period) is defined for each vector-virus combination. For dengue, average of 7–12 days reported, but could be as early as 4 days.	Immediate transmission at next blood meal once viruses have infected salivary glands.	Watts et al., 1987, Salazar et al., 2007, Kilpatrick et al., 2008
		Flaviridae	Zika, Yellow fever, Dengue, West Nile, etc			
		Bunyaviridae	La Cross, Rift Valley			
	Protozoans	*Plasmodium* sp	Malaria	Only sexual stages (gametocytes) survive in vector. Fertilization results in an ookinete that moves out of the midgut lumen and settles in the midgut outer epithelium. Transforms into an oocyst. Asexual multiplication occurs (sporogony). Cyst opens and sporozoites migrate to salivary glands through hemocoel. They are imbedded into a parasitophorus vacuole until they are released into the salivary ducts. About two weeks are needed from ingestion of gametocytes to migration of sporozoites to salivary glands. Timing dependent on parasite-mosquito species combination.	Immediate transmission at next blood meal once sporozoites are present in the salivary ducts.	Meis et al., 1992, Vlachou et al., 2006
	Nematodes (Filariae)	*Wuchereria bancrofti* *Brugia malayi*	Lymphatic filariasis	Ingested microfilariae (mf) cross the vector midgut wall to enter the thoracic muscles. Subsequent molting to L1, L2 and to the L3 infective stage. L3 migrate back into the hemocoel, then to the head and mouth parts. . No active injection by vector. L3 penetrate the host skin at biting site. Development from mf to L3 takes at least 10–11 days.	Immediate transmission at next blood meal once L3 have reached the mosquito mouthparts.	Paily et al., 2009
Sand flies (Phlebotominae) *Lutzomyia* sp *Phlebotomus* sp	Protozoans	*Leishmania sp Subgenera *Leishmania* sp *Vannia* (New World only)	Cutaneous and visceral leishmaniasis	Amastigotes (intracellular in host macrophages) taken up mainly from skin at insect bite. Changes within the vector's internal environment triggers the transformation into motile procyclic promastigotes that multiply in bloodmeal. After few days, differentiation into highly motile elongated nectomonad promastigotes. *Leishmania*: nectomonad promastigotes migrate to the anterior part of the midgut and break out of the peritrophic membrane. They move to the cardia and transform into leptomonad promastigotes which further multiply and produce a promastigote secretory gel. Some attach and transform into haptomonad promastigotes. Some differentiate into infective metacyclic promastigotes. *Vannia*: Similar to *Leishmania*, except a concentration and replication step in hindgut. Attachment as haptomonad promastigotes. Migration to foregut and establishment in the cardia as leptomonad promastigotes. Both subgenera: The gel containing the infective metacyclic forms obstructs the anterior midgut, forcing regurgitation at next bite prior to feeding, releasing the pathogen into the host. One-2 weeks are needed between ingestion of amastigotes and regurgitation of the infective metacyclic promastigotes.	Immediate transmission at next bite once the gel containing the pathogens is blocking the foregut of the vector, in a way that the vector has to expel the gel into the host to be able to feed.	Killick-Kendrick, 1990, Bates, 2007
Black flies (Simuliidae) *Simulium* sp	Nematodes (Filariae)	*Onchocerca volvulus*	River blindness	Ingested microfilariae (mf) cross the vector midgut wall to enter the thoracic muscles. Subsequent molting to L1, L2 and to the L3 infective stage. L3 migrate back to the hemocoel, then to the head and mouth parts. . No active injection by vector. L3 penetrate the host skin at biting site. Development in vector takes 6–11 days, depending on temperature and vector species. The simulids need to feed 1–2 times before the infective L3 are fully developed.	Immediate transmission at next blood meal once L3 have reached the vector mouthparts.	World Health Organization, 1976, Takaoka et al., 1982
Biting midges (*Ceratopogonidae*) *Culicoides* sp.	Nematodes (Filariae)	*Mansonella perstans*	Mansonellosis	Life-cycle similar to *O. volvulus.* Development in vector takes 7–9 days.	Immediate transmission at next blood meal once L3 have reached the vector mouthparts.	Simonsen et al., 2011
Tsetse flies (*Glossinidae*) *Glossina* sp	Protozoans	*Trypanosoma gambiense*, *T. rhodesesiense*	African trypanosomiasis	Ingestion by vector of bloodstream trypanosomes. Transformation into procyclic trypomastigotes and intense multiplication in midgut from day 3 after feeding. From day 6 migration starts from hindgut to foregut, pharynx and finally salivary glands. Metacyclic trypomastygotes are the infective stage, detectable in salivary glands from day 12 after feeding and can be injected at next blood meal. Flies infective for the rest of their lives.	Immediate transmission at next blood meal once infective metacyclic forms are developed in salivary glands.	Van Den Abbeele et al., 1999, Gibson and Baileys, 2003
Tabanids (Tabanidae) *Chrisops* sp	Nematodes (Filariae)	*Loa loa*	African eyeworm	Ingested mf exsheath in midgut and migrate predominantly to abdominal fat bodies. Subsequent molting to L1, L2 and then to the infective L3. L3 migrate back into the hemocoel, then to the head and mouthparts. Development time is temperature dependent, requiring 7–10 days.	Immediate transmission at next blood meal once L3 have reached the vector mouthparts.	Orihel and Lowrie, 1975
True bugs (Hemiptera, Reduviidae) *Rhodnius* sp *Triatoma* sp	Protozoans	*Trypanosoma cruzi	Chagas disease	Bloodstream trypomastigotes ingested by vector. Change to spheromastigotes and then to epimastigotes. Active multiplication in hindgut. Transformation into infective metacyclic forms, released with feces or Malpighian secretions. Infection via rubbing feces over skin lesions, contact with mucosae (mouth, nose, eye), or ingestion of the whole bug. Development timing is temperature and vector species dependent, At least 15–30 days are needed to detect infective metacyclic forms in the hindgut. Timing is shorter in immature instars (6–15 days).	Immediate transmission once infective forms are present in feces.	Perlowagora-Szumlewicz and de Carvalho Moreira, 1994, Krinsky, 2008, Bern et al., 2011
Fleas (Siphonaptera, Pulicidae) *Xenopsylla* and *Pulex* sp (plague) *Ctenocephalides* sp	Bacteria	*Yersinia pestis*	Plague	After ingestion, bacteria multiply in the midgut until the total blockage of the flea proventricule is achieved. Host infection occurs via regurgitation by the flea, or via direct contact and aerosol during epidemic. In the flea vector, 4–16 days are needed to complete proventricule blockage. Depending on temperature and flea species, timing to reach proventricule blockage can be much longer.	Immediate transmission at next bite once proventricule blockage is achieved.	Gage and Kosoy, 2005
		*Rickettsia felis*	Cat flea typhus	Transmitted by *C. felis*. Ingestion by feeding on an infected host. Multiplication in midgut cells and dissemination in the flea tissues, including ovaries and salivary glands. Migration to salivary glands takes 7–14 days but transmission has been reported to occur as soon as 12 h after infection feeding (surely within 24 h)via co-feeding with infected fleas. This early phase transmission seems to be mechanical. Transovarial transmission also occurs in the flea vector. Mosquitoes (*Anopheles gambiense*) now also suspected to be vector.	Between 12 and 24 h for transmission via co-feeding. Timing for infection in host not measured per se. Could be immediate at next bite once salivary glands are invaded.	Thepparit et al., 2013, Brown et al., 2016, Angelakis et al., 2016
		*Bartonella henselae	Cat scratch disease	Transmitted by *C. felis*. Pathogen ingested via an infected blood meal. Acquisition starts 3 h after feeding begins. Replication occurs in gut cells. Bacteria survive during entire flea life-span. Detected in feces 24 h after 1st feeding starts. Survival in flea feces estimated to be at least 3 days. Host infection through exposure with flea feces, ingestion of infected fleas or flea feces, scratching or biting of a flea contaminated carrier animal.	Immediate transmission via exposure to contaminated feces. In unfed fleas starting to feed, 24 h delay before infected feces are released.	Bouhsira et al., 2013
Lice (Phthiraptera, Pediculidae) *Pediculus* sp	Bacteria	*Rickettsia prowazekii*	Epidemic typhus	The pathogen develops in gut cells and is released in the gut lumen when the cells break. The insect feces are infectious. Host infection occurs when the skin damaged by scratching comes into contact with infected feces A minimum of 5 days required between feeding on infected blood and first release in feces. Narrow infection time window as infected lice die prematurely due to gut cell burst and perforation.	Immediate transmission if infected feces in contact with wounds due to scratching.	Houhamdi et al., 2002
		*Bartonella quintana*	Trench fever	The pathogen multiplies in gut lumen and in epithelial gut cells, then shed in feces. Infection via skin damaged by scratching, contact with eyes mucosa, or if wounds are in contact with contaminated bedding or clothes. Five to 8 days needed between feeding on an infected host and detection of the pathogen in lice feces.	Immediate transmission if infected feces in contact with wounds due to scratching.	Byam and Lloyd, 1920, Fournier et al., 2001
Hard ticks (Ixodidae) *Amblyomma* sp *Dermacentor* sp *Ixodes* sp, *Ripicephalus* sp *Hyalomma* sp *Haemaphysalis* sp	Arboviruses	*Bunyaviridae	Crimean-Congo hemorrhagic fever (old world)	Transmitted by *Hyalomma* sp. Virus persistence in the vector through transstadial and transovarial transmission. Venereal transmission from males to female ticks also occurs. Intrastadial virus transfer via co-feeding demonstrated. Development time in vector not measured. Contamination also reported via direct contact with an infected host, raw meat or milk ingestion, aerosol etc.	Immediate transmission at next blood meal once viruses have reached and multiplied in salivary glands.	Nuttall et al., 1994, Charrel et al., 2004
			Heartland virus (USA)	*Amblyomma americanum* seem to be main vector. Virus persistence in the vector through transstadial and transovarial transmission. Intrastadial virus transfer via co-feeding demonstrated. Development in vector not described in details. Virus detected in midgut epithelial cells after infection feeding.	Transmission timing not reported, but likely to be similar to other Bunyaviridae.	Godsey et al., 2016
		Flaviridae	Tick-borne encephalitis (TBE)	Transmitted by *Ixodes* sp. Virus persistence in the vector through transstadial and transovarial transmission. Virus amplification via co-feeding on infected reservoirs. Virus multiplication takes place first in gut cell, then other tissue cells are invaded with further replication. Salivary glands are invaded relatively late, probably after molting as this organ undergoes resorption and regeneration during molting. Virus detected in salivary glands before the next the blood meal starts. Transmission cases through infected milk and derivate are also reported.	Transmission occurs presumably as soon as feeding starts, as salivary glands are invaded prior to feeding.	Nuttall et al., 1994, Karbowiak and Biernat, 2016
	Bacteria	*Rickettsia conorii* *R. rickettsii*	Fièvre boutonneuse Rocky Mountain spotted fever	*R. conorii* transmitted by many tick genera*. R. rickettsii* transmitted by *Dermacentor* sp. The pathogen can multiply in almost all organs of the vector. If present in ovaries, transovarial transmission can occur. Persistence in the vector also through transstadial transmission. The pathogen is avirulent in ticks that have not fed for a long time period. Reactivation can be triggered by the temperature increase that typically occurs during blood feeding on a warm-blooded vertebrate.	At least 10 h of tick feeding are needed before the pathogen becomes infective again and can be successfully transmitted.	Hayes and Burgdorfer, 1982
		*Anaplasma phagocytophylum	Human granulocytic anaplasmosis	Transmitted by *Ixodes* sp. Persistence in the vector through transstadial transmission, but not transovarial. Acquisition by the vector within 24 h of blood feeding. Multiplication in vector during and after acquisition feeding, and triggered again by next blood meal.	Transmission does not take place before 36 h-48 h post tick feeding. In the lab, transmission has occasionally been shown to occur within 24 h of attachment.	Hodzic et al., 1998, Katavolos et al., 1998, des Vignes et al., 2001
		*Ehrlichia ewingii	Human granulocytic ehrlichiosis	Transmitted by *Amblyomma americanum*. Life cycle in ticks not described. Closely related to *E. chaffeensis*. Development time in vector not measured. In one transmission study, pathogens were detected in host only 11–28 days after the beginning of exposure to adult ticks having acquired infection at the nymph stage. The speed of detection is depending on the size of the inoculum.	Timing not measured.	Paddock and Childs, 2003, Yabsley et al., 2011
		*Ehrlichia chaffeensis	Human monocytic ehrlichiosis	Transmitted by *A. americanum* nymphs and adults. Persistence in the vector through transstadial transmission, not transovarial. Development time in vector not measured. In one transmission study, pathogens were detected in host only 7–12 days after the beginning of exposure to adult ticks having acquired infection at the nymph stage.	Timing not measured.	Paddock and Childs, 2003, Varela-Stokes, 2007
		*Borrelia burgdorferi sensu lato complex	Lyme disease	Transmitted by *Ixodes* sp. Persistence in the vector through transstadial transmission, not transovarial. After ingestion of infected blood, the spirochetes multiply in the tick's midgut by binary fission. They survive the vector molting. They migrate from the tick midgut to the salivary glands within 24 h after the start of the transmission feeding.	Most transmission occurs between 48 h and 72 h after tick attachment. But some studies report infection as early as 16 h post attachment.	Kahl et al., 1998, des Vignes et al., 2001
	Protozoans	*Babesia divergens	Human babesiosis (EU)	*Ixodes ricinus* is the main vector. Persistence in the vector through transovarial and transstadial transmission. Only adult ticks seem to be able to acquire infection, but all stages can transmit. The sexual part of the life-cycle occurs in the vector. Ingested gametocytes fuse to give rise to immobile zygotes that transform into mobile kinetes. They enter the hemolymph, disseminate into various tissues including muscles, epidermis, Malpighian tubules and ovaries in adults. They undergo an additional asexual multiplication step and further dissemination as secondary kinetes. In salivary glands, kinetes continue to multiply asexually. The maturation into infective haploid sporozoites happens only after transmission feeding starts. In nymph ticks, sporozoites were detected in salivary glands from the 3rd day of feeding.	Like other *Babesia*, transmission is delayed to the second half of the tick blood meal. Transmission reported from day 3 of feeding.	Donnelly and Pierce, 1975, Homer et al., 2000, Zintl et al., 2003
		*Babesia microti	Human babesiosis (US)	Transmitted by *Ixodes* sp. Persistence in the vector through transstadial transmission over one stage only, not transovarial. Nymphs are the main transmission stage. The sexual part of the life-cycle occurs in the vector. The ingested gametocytes fuse into zygotes that move to the gut epithelial cells, then to the hemolymph as ookinetes and reach the salivary glands. They establish and become a multinucleate sporoblast after asexual multiplication. Maturation of infective sporozoites starts only after the tick host begins to feed again.	Transmission success increases with feeding time. Minimal infectious dose of sporozoites detected 48 h after transmission feeding starts. Maximum numbers of sporozoites found in salivary glands 60 h after feeding start.	Piesman and Spielman, 1982, Homer et al., 2000, Gray et al., 2002
Hard and soft ticks (Ixodidae and Argasidae)	Bacteria	*Coxiella burnetti	Q fever	Transmitted by many tick genera. Persistence in the vector through transovarial and transstadial transmission. Multiplication in midgut cells. The bacteria are released in tick feces when the tick begins to feed again. Transmission via an arthropod vector is very rare; occurs mostly through aerosol or from parturient fluids released by infected vertebrate hosts. The pathogen persists in the environment for weeks and can be spread by the wind.	Timing not known in feeding ticks.	Maurin and Raoult, 1999
Soft ticks (Argasidae) *Ornithodoros* sp.	Bacteria	*Borrelia duttoni (old world);* *B. hermsii, B, turicatae, B. parkeri (new world)*	Tick-borne relapsing fever	*Ornithodoros moubata* (*B. duttoni*): after ingestion with the bloodmeal, the pathogens enter the hemolymph and invade numerous tissues including synganglion, salivary glands, ovaries and coxal organs. Transmission via saliva in nymph ticks, and mainly via coxal fluid contamination of tick bite in adult ticks. Persistence in the vector through transovarial and transstadial transmission. *O. hermsi* (*B. hermsii*): Transmission via saliva in all tick instars. Persistence in vector mainly through transstadial transmission, very rare transovarial transmission reported.	Ticks: Transmission can occur within minutes, and has even been shown happening as quickly as 30 s after tick bite.	Schwan and Piesman, 2002, Schwan and Piesman, 2002, Dworkin et al., 2008
Lice (Phthiraptera, Pediculidae) *Pediculus humanis*		*Borrelia recurrentis*	Lice-borne relapsing fever	Ingestion by feeding on an infected host. From the midgut, spirochetes infect the body cavity and multiply without invading other tissues. No transovarial transmission. Transmission occurs when lice are crushed during scratching and spirochete-infected hemolymph is released onto the host skin.	Immediate.	Piesman and Schwan, 2010
Chigger mites (Trombiculidae) *Leptotrombidium* sp	Bacteria	*Orientia tsutsugamushi*	Oriental scrub typhus	Chiggers feed only once on infected host. Only larvae are parasitic. Chiggers do not feed on blood. They inject digestive fluids to digest the host's tissues and feed on serum exudates. Details on the pathogen life-cycle in the vector not described. The pathogen is likely to be inoculated into the extra-cellular exudates during feeding. Persistence in the vector mainly through transovarial transmission. Transstadial and co-feeding transmission have also been shown.	Timing not known.	Lerdthusnee et al., 2002
Tabanids, mosquitoes, fleas, hard ticks	Bacteria	*Francisella tularensis	Tularemia	Main ways of transmission via tick bites and direct contact with a contaminated animal, mainly rabbits and hares, but occurs also via insect bites, ingestion of contaminated food or aerosol. Ticks: *Dermacentor variabilis* is the main vector. Persistence in the vector through transstadial transmission, although infected nymph ticks suffer high mortality due to the pathogen. Transovarial transmission also reported.	Ticks: can occur within 1 day after an adult tick infected as nymph begins to feed.	Reese et al., 2011

**Table 2:** Companion animal vector-transmitted pathogens (*zoonotic diseases).

Vector	Pathogen class		Disease	Pathogen development phases and timing in vector	Pathogen- to- host transfer timing at vector bite	References
Hard ticks (Ixodidae) *Rhipicephalus* sp *Dermacentor* sp *Haemaphysalis* sp *Amblyomma* sp *Ixodes* sp	Bacteria	*Ehrlichia ewingii	Canine granulocytic ehrlichiosis	Transmitted by *Amblyomma americanum*. Life cycle in ticks is not described. Closely related to *E. chaffeensis*. Development time in vector not measured. In one transmission study, the pathogens were detected in host only 11–28 days after the beginning of exposure to adult ticks having acquired infection at the nymph stage. The speed of detection is dependent on the size of the inoculum.	Timing not measured.	Paddock and Childs, 2003, Yabsley et al., 2011
		*Ehrlichia chaffeensis	Canine monocytic ehrlichiosis	Transmitted by *A. americanum* nymphs and adults. Persistence in vector through transstadial transmission, not transovarial. Development time in vector not measured. In one transmission study, pathogen detected in host only 7–12 days after beginning of exposure to adult ticks having acquired infection at the nymph stage.	Timing not measured.	Paddock and Childs, 2003, Varela-Stokes, 2007
		*Ehrlichia canis*	Canine ehrlichiosis	*Rhipicephalus sanguineus* is the main vector. *D. variabilis* also reported to be vector. Development in ticks not investigated in details. Pathogen likely to multiply within gut cells, hemocytes and salivary glands. Persistence in the vector through transstadial transmission. Intrastadial infection reported (infection amongst ticks of same stage co-feeding). Importance of male ticks in the epidemiology of the disease as they can move from host to host and could transmit the pathogen more efficiently.	Host infection can occur as soon as 3 h post tick attachment.	Stich et al., 2008, Fourie et al., 2013b
		*Anaplasma platys*	Canine cyclic thrombocyto-penia	*R. sanguineus* is the main vector. Rickettsia-like organism. Life-cycle in ticks not described.	Timing not measured, could be within 2 days, likely hours.	Dantas-Torres et al., 2013
		*Borrelia burgdorferi sensu lato complex	Lyme disease	Transmitted by *Ixodes* sp. Persistence in the vector through transstadial transmission, not transovarial. After ingestion of infected blood, spirochetes multiplies in the tick's midgut by binary fission. They survive the vector molting. They migrate from the midgut to the salivary glands within 24 h after the start of the transmission feeding.	Most transmission occurs between 48 h and 72 h after tick attachment. But some studies report infection as early as 16 h post attachment.	Kahl et al., 1998, des Vignes et al., 2001
	Protozoans	*Babesia canis (EU), B. vogeli (USA)*	Canine babesiosis	*R. sanguineus* (US, EU), *D. reticulatus* (EU), and *H.leachi* (Africa) are reported to be vectors. Persistence in the vector through transovarial and transstadial transmission. The sexual part of the life-cycle occurs in the vector. Ingested gametocytes fuse into zygotes, transform into mobile kinetes that enter the hemolymph and disseminate into various tissues including muscles, epidermis and Malpighian tubules. They undergo a further division cycle. Secondary ookinetes invade almost all tick tissues, including ovaries in adults and salivary glands. In salivary glands, asexual multiplication by binary fission occurs. The maturation into infective sporozoites happens only after transmission feeding starts. Sporozoites formation in salivary glands takes 2–3 days.	At least 48 h of feeding are needed before transmission occurs. But if male ticks have already been feeding once, transmission could be immediate on the next host visited.	Schein et al., 1979, Mehlhorn and Schein, 1984, Heile et al., 2007
		*Babesia microti	Canine babesiosis	Transmitted by *Ixodes* sp. Persistence in the vector through transstadial transmission over one stage only, not transovarial. Nymphs are the main transmission stage. The sexual part of the life-cycle occurs in the vector. The ingested gametocytes fuse into zygotes that move to the gut epithelial cells, then to the hemolymph as ookinetes and reach the salivary glands. They establish and become a multinucleate sporoblast after asexual multiplication. Maturation of infective sporozoites starts only after the tick host begins to feed again.	Transmission success increases with feeding time. Minimal infectious dose of sporozoites reported 48 h after transmission feeding start. Maximum numbers of sporozoites detected 60 h after feeding start.	Piesman and Spielman, 1982, Homer et al., 2000, Gray et al., 2002
		*Babesia vulpes* (= *Theileria annae*)		Tick vector not known but likely to be *Ixodes* sp. and *R. sanguineus*. Life-cycle in vector not described but likely to be similar to *B. microti* as genetically closely related to it.	Timing not measured, but likely to be delayed like other *Babesia* sp.	Baneth et al., 2015
		*Hepatozoon canis*	Canine hepatozoonosis	*R. sanguineus* is the main vector. Infection by ingestion of gamonts from an infected dog. Persistence in the vector through transstadial transmission, not transovarial. Mature oocysts containing infective sporozoites located in the hemocoel. Dogs get infected through oral ingestion of a tick containing oocysts. Oocysts break inside the dog's digestive system, releasing the infective sporozoites. Transmission success is more dependent on temperature more than blood meal size dependent. Success is higher if the tick has been feeding for some days, being heated by the ingested blood, but transmission also works when unfed ticks are ingested.	Immediate transmission.	Baneth et al., 2001
		*Cytauxzoon felis*	Cat theileriosis	*A. americanum* is the main vector. Persistence in the vector through transstadial transmission. Details of the life-cycle in vector not reported.	Transmission occurs within 48 h feeding.	Reichard et al., 2013
Mosquitoes (Culicidae) *Aedes* sp *Ochlerotatus* sp *Anopheles* sp *Culex* sp	Nematodes (Filariae)	*Dirofilaria immitis*	Heartworm	Ingested microfilariae (mf) cross the vector midgut wall to enter the Malpighian tubules. Subsequent molting to L1, L2 and to the L3 infective stage. L3 migrate back to the hemocoel, then to the head and mouth parts. No active injection by vector. L3 penetrate the host skin at biting site. Development from mf to L3 lasts about 15 days–17 days (*Aedes aegypti*), temperature and mosquito species dependent.	Immediate transmission at next blood meal once L3 have reached the mosquito mouthparts.	Taylor, 1960, Montarsi et al., 2015
Sand flies (Phlebotominae) *Lutzomyia* *Phlebotomus*	Protozoans	*Leishmania sp.	Cutaneous and visceral leishmaniasis	Amastigotes (intracellular in host macrophages) taken up mainly from skin at insect bite. Changes within the vector's internal environment triggers the transformation into motile procyclic promastigotes that multiply in bloodmeal. After few days, differentiation into highly motile elongated nectomonad promastigotes. They migrate to the anterior part of the midgut and break out of the peritrophic membrane. They move to the cardia and transform into leptomonad promastigotes which further multiply and produce a promastigote secretory gel. Some attach and transform into haptomonad promastigotes. Some differentiate into the infective metacyclic promastigotes. The gel containing the infective metacyclic forms obstructs the anterior midgut, forcing regurgitation at next bite prior to feeding, releasing the pathogen into the host. One-2 weeks are needed between ingestion of amastigotes and regurgitation of the infective metacyclic promastigotes.	Immediate transmission at next bite once the gel containing the pathogens is blocking the foregut of the vector.	Killick-Kendrick, 1990, Bates, 2007
True bugs (Hemiptera, Reduviidae) *Rhodnius* sp	Protozoans	*Trypanosoma cruzi	Chagas disease	Bloodstream trypomastigotes ingested by vector. Change to spheromastigotes and then to epimastigotes. Active multiplication in hindgut. Transformation into infective metacyclic forms, released with feces or Malpighian secretions. Infection via rubbing feces over skin lesions, contact with mucosae (mouth, nose, eye), or ingestion of the whole bug. Development timing is temperature and vector species dependent. At least 15–30 days are needed to detect infective metacyclic forms in the hindgut. Timing is shorter in immature instars (6–15 days).	Immediate transmission once infective forms are present in feces.	Perlowagora-Szumlewicz and de Carvalho Moreira, 1994, Krinsky, 2008, Bern et al., 2011
Fleas (Siphonaptera, Pulicidae) *Ctenocephalides* sp	Bacteria	*Bartonella henselae	Cat scratch disease	Transmitted by *C. felis*. Pathogen ingested via an infected blood meal. Acquisition starts 3 h after feeding begins. Replication occurs in gut cells. Bacteria survive during entire flea life-span. Detected in feces 24 h after 1 s t feeding starts. Survival in flea feces estimated to be at least 3 days. Host infection through exposure with flea feces, ingestion of infected fleas or flea feces, scratching or biting of a flea contaminated carrier animal.	Immediate transmission via exposure to contaminated feces. In unfed fleas starting to feed, 24 h delay before infected feces are released.	Bouhsira et al., 2013
Fleas (Siphonaptera, Pulicidae) *Ctenocephalides* sp Lice (Phthiraptera, Trichodectidae) *Trichodectes* sp	Cestodes	*Dipilidium caninum*	Dog tapeworm	Vector gets infected at larval stage through cestode egg ingestion. Development in fleas is temperature dependent. With temperature lower than 30 °C, the infective metacestode is not ready when the adult fleas emerge. The flea will need to survive and stay on a host a few days to allow completion of the development of the metacestode, triggered by the higher temperature of the host. Blood meal has no effect on development. Dog infection through ingestion of the parasitized flea.	Immediate transmission once the infective larvae is mature.	Pugh, 1987

**Table 3:** Farm animal vector-transmitted pathogens (* zoonotic diseases).

Vector	Pathogen class		Disease	Pathogen development phases and timing in vector	Pathogen- to- host transfer timing at vector bite	References
Hard ticks (Ixodidae) *Rhipicephalus* sp *Dermacentor* sp *Amblyomma* sp *Ixodes* sp *Hyalomma* sp	Viruses	*Bunyaviridae	Crimean-Congo hemorrhagic fever	Transmitted by *Hyalomma* sp. Virus persistence in the vector through transstadial and transovarial transmission. Venereal transmission from males to female ticks also occurs. Transmission via co-feeding demonstrated. Development time in vector not measured. Contamination can also occur via direct contact with an infected host, raw meat or milk ingestion, aerosol etc.	Immediate transmission at next blood meal once viruses have reached and multiplied in the salivary glands.	Nuttall et al., 1994, Charrel et al., 2004
		*Flaviviridae	Louping ill	Transmitted by *Ixodes ricinus*. Virus persistence in the vector through transstadial transmission, no evidence of transovarial transmission. Closely related to TBE virus. Transmission between vectors via co-feeding demonstrated. Development time in vector not measured.	Immediate transmission at next feeding once viruses have reached and multiplied in salivary glands.	Jeffries et al., 2014
	Bacteria	*Anaplasma phagocytophylum	Granulocytic anaplasmosis	Transmitted by *Ixodes* sp. Persistence in the vector through transstadial transmission, but not transovarial. Acquisition by the vector within 24 h blood feeding. Multiplication in vector during and after acquisition feeding, and triggered again by next blood meal.	Transmission does not take place before 36 h-48 h tick feeding, but was shown in the lab to occasionally occur occasionally within 24 h of attachment already.	Hodzic et al., 1998, Katavolos et al., 1998, des Vignes et al., 2001
		*Ehrlichia* (= *Cowdria*) *ruminantium*	Heartwater	Transmitted by *Amblyomma* sp. Persistence in the vector through transstadial transmission. Transstadial transmission can happen over one or more stages depending on tick species. No interstadial transmission reported and transovarial transmission not sure. Bacteria ingested with the blood meal and enter the gut cells into which they multiply by binary fission in inclusion bodies. Migration to other organs like hemocytes, Malpighian tubules and salivary glands. Bacteria colonies detected in salivary glands only after transmission feeding start.	Transmission reported to occur from the 2nd day of feeding in nymphs, and from the 4th day in adult ticks.	Kocan and Bezuidenhout, 1987, Bezuidenhout, 1987, Allsopp, 2010
		*Borrelia theileri*	Bovine borreliosis	Transmitted by *Rhipicephalus microplus*. Persistence in the vector through transstadial and transovarial transmission. Spirochetes ingested or present in ticks, multiply in hemocytes and hemolymph, with a tropism for ovaries and central ganglion. Also transitory presence of the bacteria can be detected in other organs. Salivary glands may be invaded prior to or during the next blood meal. Larvae do not transmit infection as spirochetes are too scarce to sufficiently invade salivary glands. The pathogen multiplication is proportional to the level of tick nutrition. Highest in adult females.	Timing not measured.	Smith et al., 1978
	Protozoans	*Babesia divergens	Bovine babesiosis (EU)	*Ixodes ricinus* is the main vector. Persistence in the vector through transovarial and transstadial transmission. Only adult ticks seem to be able to acquire infection, but all stages can transmit. The sexual part of the life-cycle occurs in the vector. Ingested gametocytes fuse to give rise to immobile zygotes that transform into mobile kinetes, They enter the hemolymph, disseminate into various tissues including muscles, epidermis, Malpighian tubules and ovaries in adults. They undergo an additional asexual multiplication step and further dissemination as secondary ookinetes. In salivary glands, kinetes continue to multiply asexually. Maturation into infective haploid sporozoites happens only after transmission feeding starts. In nymph ticks, sporozoites were detected in salivary glands from the 3rd day of feeding.	Like other *Babesia*, transmission is delayed to the second half of the tick blood meal. Transmission reported from day 3 of feeding.	Donnelly and Pierce, 1975, Homer et al., 2000, Zintl et al., 2003
		*Babesia bovis B. bigemina*	Bovine babesiosis (ROW)	Transmitted by *Rhipicephalus (Boophilus) sp.* that are one-host ticks. Persistence in the vector through transovarial and transstadial transmission. The sexual part of the life-cycle occurs in the vector. Ingested gametocytes develop into gametes (ray bodies) that fuse to form diploid zygotes andenter the gut cells. Asexual multiplication and generation of kinetes that will migrate to invade other tissues, including salivary glands and tick’ oocytes. A secondary multiplication step occurs in these tissues. In salivary glands, kinetes transform into multinucleate stages that break up to form infective sporozoites. Maturation into infective sporozoites will start only when the tick is feeding again. *B. bigemina*: 9 days required for the development of infective sporozoites. Transmission possible only at nymph and adult stage. *B. bovis*: infective stages detected 2–3 days after feeding starts. Larvae can also transmit infection.	In tick larvae, transmission delayed due to maturation of sporozoites taking place after tick feeding starts. Nine days needed, in *B. bigemina*, 2–3 days in *B. bovis.*	Bock et al., 2004, Howell et al., 2007
		*Theileria parva*	East Coast fever	*Rhipicephalus appendiculatus* is the main vector for *T. parva*. *T. annulata* and *T. lestoquardi* transmitted by *Hyalomma* sp. Persistence in the vector trough transstadial transmission only. The sexual part of the life-cycle occurs in vector. Ingested merozoites undergo gamogony. The resulting gametocytes fuse into zygotes that enter the gut cells. Maturation into ookinetes without obvious multiplication. Ookinetes move out the gut cells and migrate to the salivary glands cells through the hemolymph. Transformation into multinucleate sporonts. Ticks are molting at this stage. Development stopped until the next blood meal begins, triggering a secondary multiplication and differentiation into infective sporozoites.	*T. annulata*: 2 days feeding required for infective sporozoites to be ready for transmission. Can take up to 6–9 days in ticks having starved for months. No information on the other species.	Schein et al., 1975, Nicholson et al., 2008
		*Theileria annulata*	Tropical theileriosis (cattle)			
		*Theileria lestoquardi*	Old world tropical theileriosis (sheep)			
Hard ticks (Ixodidae) Tabanids, stable flies, mosquitoes	Bacteria	*Anaplasma marginale*	Cattle anaplasmosis, erythrocytic anaplasmosis	Ticks: transmitted by many tick genera. Persistence in the vector through interstadial and transstadial transmission. No transovarial transmission. Importance of male ticks (i.e Dermacentor sp) that remain persistently infected and feed on different hosts. Acquisition by the vector within 24h blood feeding. Multiplication in gut cells, then in other tissues, including salivary glands. Multiplication triggered again by next blood meal. Insects: mechanical transmission via contact with infected mouthparts. Transmission also via contaminated fomites (i.e. needles).	Ticks: transmission does not take place before 36 h-48 h tick feeding, but was shown in the lab to occasionally occur after 24 h.	Kocan et al., 2004, Kocan et al., 2010
Hard and soft ticks (Ixodidae and Argasidae)		*Coxiella burnetti	Q fever	Transmitted by many tick genera. Persistence in the vector through transovarial and transstadial transmission. Multiplication in midgut cells. The bacteria are released in tick feces when the tick begins to feed again. Transmission via an arthropod vector is very rare, occurs mostly through aerosol or from parturient fluids released by infected vertebrate hosts. The pathogen persists in the environment for weeks, and can be spread by the wind.	Timing not known in feeding ticks.	Maurin and Raoult, 1999
Tsetse flies (Glossinidae) *Glossina* sp Tabanids, stable flies (*T. vivax*)	Protozoans	*Trypanosoma brucei,* *T. congolense,* *T. vivax*	Nagana (cattle) Sheep trypanosomiasis (*T. vivax*)	General life-cycle in vector similar to human trypanosomes (Table 1). *T. brucei*: 12 days to 2 weeks needed to complete the cycle in flies. Metacyclic forms found in salivary glands. Flies remain infective for life afterwards. *T. congolense*: do not infect salivary glands. Epimastigotes attach to the labrum and hypopharynx of the proboscis, proliferate and mature into infective metacyclic forms. *T. congolense*: infective metacyclic forms detected in saliva 21 days after infection feeding. Flies remain infective straight at bite afterwards. *T. vivax*: after ingestion, only elongated forms survive in foregut and migrate to the cibarium. They transform into epimastigotes that migrate to the proboscis, attach and multiply. Free pre-metacyclic forms are generated by asymmetric fission of the epimastigotes. Host infection process not clear: either pre-metacyclic forms invade the hypopharynx and differentiate into infective metacyclic forms and/or metacyclic forms remaining in the alimentary canal are expelled via regurgitation before blood meal ingestion. At least 3 days are needed from infection feeding to detect infective forms in the hypopharynx. In addition to transmission by tsetse flies, mechanical transmission by other biting flies like stable flies and tabanids also occurs. Intabanids, the pathogen can survive in the crop or midgut up to 5–7 h, and be regurgitated during early stages of feeding.	Immediate transmission at next blood meal once infective metacyclic forms have matured.	Gibson and Baileys, 2003, Peacock et al., 2012, Desquesnes et al., 2013, Ooi et al., 2016
Tabanids (Tabanidae) *Tabanus* sp*, Chrysops* sp Stable flies (Muscidae) *Stomoxys* sp	Protozoans	*Trypanosoma evansi*	Surra	Mechanical transmission of the pathogen during restart of feeding on a different host after feeding interruption on an infected host. Infected blood remains in mouth parts and is reinjected with saliva into the next host. Blood meal regurgitation also shown. Trypanosomes survive up to 30 min on mouthparts. Survival in midgut can be hours, up to 48 h in *Stomoxys* flies allowing pathogen regurgitation in a delayed transmission mode.	Immediate transmission at insect bite.	Desquesnes et al., 2013
Black flies (Simuliidae) *Simulium* sp Biting midges (Ceratopogonidae) *Culicoides* sp	Nematodes (Filariae)	*Onchocerca lienalis,* *O. ochengi,* *O. dukei,* *(O. gutturosa)* *O. gibsoni, O. gutturosa*	Bovine onchocercosis	Ingested microfilariae (mf) cross the vector midgut wall to enter the thoracic muscles. Subsequent molting to L1, L2 and to the L3 infective stage. L3 migrate back into the hemocoel, then to the head and mouth parts. No active injection by vector. L3 penetrate the host skin at biting site. Development time to infective L3 is temperature dependent: *O. ochengi* in about 6 days, *O. dukei* in 6–9 days, *O. gibsoni* in about 6 days at 30 °C, *O. gutturosa* in 13–15 days at 23 °C, but up to 19 days have been reported. *O. lienalis* similar to *O. gutturosa*.	Immediate transmission at next blood meal once L3 have reached the insect mouthparts.	Eichler, 1973, Kettle, 1995
Biting midges (Ceratopogonidae) *Culicoides* sp	Viruses	Reoviridae	Bluetongue	To be transmitted, the virus ingested need to enter the midgut cells, replicate in them, escape into the hemocoel, and finally invade and replicate into the salivary glands. Development is temperature, virus serotype and vector species dependent. The EIP varies from 4 to more than 20 days. Vector remain infective for life.	Immediate transmission once salivary glands are infected.	Wittmann et al., 2002, Wilson and Mellor, 2009
Face flies (Muscidae) *Musca automnalis*	Bacteria	*Moraxella bovis*	Bovine keratocon-junctivitis	Transmission via direct contact, though feces or regurgitation of the bacteria by the vector. Regurgitation seems to play a major role. Bacteria accumulate in the fly crop.	Immediate transmission, with success depending on fly numbers feeding at same time.	Glass and Gerhardt, 1984
Tabanids, mosquitoes, fleas, hard ticks	Bacteria	*Francisella tularensis	Tularemia	Main ways of transmission via tick bites and direct contact with a contaminated animal, mainly rabbits and hares, but occurs also via insect bites, ingestion of contaminated food or aerosol. Ticks: *Dermacentor variabilis* is the main vector. Persistence in the vector through transstadial transmission although infected nymph ticks suffer high mortality due to the pathogen. Transovarial transmission also reported.	Ticks: can occur within 1 day after an adult tick infected as nymph begins to feed.	Reese et al., 2011
